# Tricarbonyl-Pyrazine-Molybdenum(0) Metal–Organic Frameworks for the Storage and Delivery of Biologically Active Carbon Monoxide

**DOI:** 10.1021/acsbiomaterials.3c00140

**Published:** 2023-03-30

**Authors:** Andreia F. Silva, Isabel B. Calhau, Ana C. Gomes, Anabela A. Valente, Isabel S. Gonçalves, Martyn Pillinger

**Affiliations:** CICECO - Aveiro Institute of Materials, Department of Chemistry, University of Aveiro, Campus Universitário de Santiago, 3810-193 Aveiro, Portugal

**Keywords:** molybdenum, pyrazine, carbon monoxide, metal−organic frameworks, CO-releasing materials

## Abstract

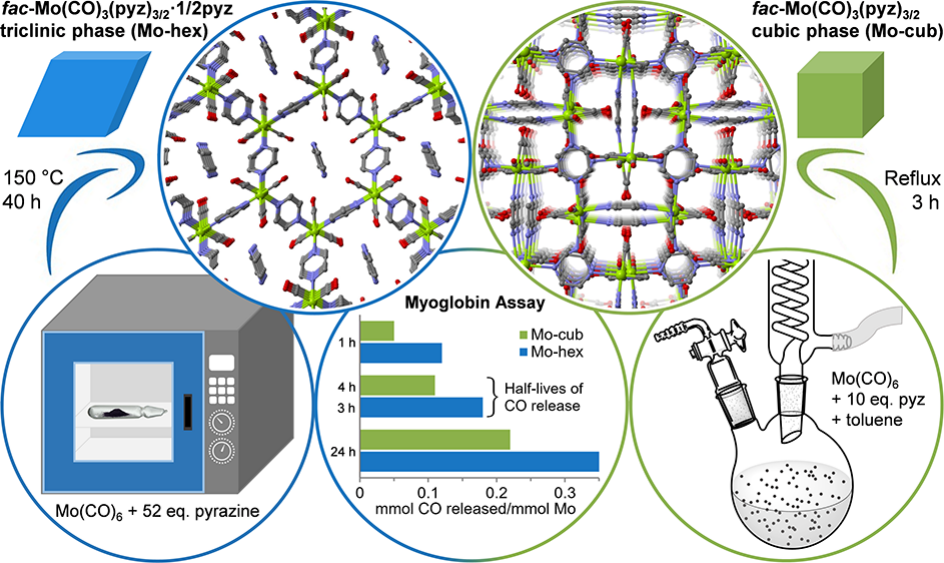

Metal–organic frameworks (MOFs) have high potential as nanoplatforms for the storage and delivery of therapeutic gasotransmitters or gas-releasing molecules. The aim of the present study was to open an investigation into the viability of tricarbonyl-pyrazine-molybdenum(0) MOFs as carbon monoxide-releasing materials (CORMAs). A previous investigation found that the reaction of Mo(CO)_6_ with excess pyrazine (pyz) in a sealed ampoule gave a mixture comprising a major triclinic phase with pyz-occupied hexagonal channels, formulated as *fac*-Mo(CO)_3_(pyz)_3/2_·1/2pyz (**Mo-hex**), and a minor dense cubic phase, formulated as *fac*-Mo(CO)_3_(pyz)_3/2_ (**Mo-cub**). In the present work, an open reflux method in toluene has been optimized for the large-scale synthesis of the pure **Mo-cub** phase. The crystalline solids **Mo-hex** and **Mo-cub** were characterized by powder X-ray diffraction (PXRD), scanning electron microscopy (SEM), thermogravimetric analysis (TGA), FT-IR and FT-Raman spectroscopies, and ^13^C{^1^H} cross-polarization (CP) magic-angle spinning (MAS) NMR spectroscopy. The release of CO from the MOFs was studied by the deoxy-myoglobin (deoxy-Mb)/carbonmonoxy-myoglobin (MbCO) UV–vis assay. **Mo-hex** and **Mo-cub** release CO upon contact with a physiological buffer in the dark, delivering 0.35 and 0.22 equiv (based on Mo), respectively, after 24 h, with half-lives of 3–4 h. Both materials display high photostability such that the CO-releasing kinetics is not affected by irradiation of the materials with UV light. These materials are attractive as potential CORMAs due to the slow release of a high CO payload. In the solid-state and under open air, **Mo-cub** underwent almost complete decarbonylation over a period of 4 days, corresponding to a theoretical CO release of 10 mmol per gram of material.

## 1 Introduction

Metal coordination compounds containing carbon monoxide (CO) and/or nitric oxide (NO) ligands are of great interest in the biomedical field as potential prodrugs for the transport and delivery of therapeutically useful amounts of gasotransmitter molecules.^1−4^ Organometallic CO-releasing molecules (CORMs) such as [Ru(CO)_3_Cl_2_]_2_ (CORM-2) have displayed numerous beneficial effects in models of cardiovascular disease, inflammatory disorders, cancer, and organ transplantation, amongst others.^5−7^ CO-release from CORMs may be triggered spontaneously through ligand exchange, endogenously, e.g., by exposure to overexpressed H_2_O_2_, enzymes or changes in pH in diseased tissues, or through an external trigger such as light or heat.

In common with other classes of small molecule drugs, the direct administration of bare organometallic CORMs may suffer from drawbacks such as low aqueous stability, nonspecific distribution throughout the body, unfavorable pharmacokinetic profiles (such as an uncontrolled, rapid release of CO in the circulation), adverse side-effects, and systemic toxicity derived from metal ions in the CORM-fragment. To address these limitations, efforts have been made to embed CO-releasing moieties into macromolecular or inorganic scaffolds.^8−14^ These CORM conjugates can potentially support a high CO payload while displaying a sustainable and controlled release of the gas, improving cellular uptake and intracellular localization, and preventing a premature interaction of the CO-releasing centers with the biological environment and limiting the circulation of decarbonylation fragments (iCORMs).

The early research on inorganic CO-releasing materials (CORMAs) centered on the surface functionalization of nonporous nanoparticles (NPs),^15−17^ e.g., a manganese tricarbonyl complex containing a modified tris(pyrazolyl)methane ligand was tethered to SiO_2_ NPs and an azide-functionalized nanodiamond.^15,16^ One difficulty encountered with this approach is that of obtaining a high degree of surface functionalization. For the CORM@SiO_2_ system, the inaccessibility of some surface azido groups limited the degree of functionalization to 22%.^15^ To achieve higher CO payloads, porous or layered materials have been studied as nanocarriers.^18−25^ The first report of this approach described the adsorption of CO on coordinatively unsaturated sites (CUS) in MIL-88B-Fe metal–organic frameworks (MOFs).^18^ When the CO-loaded MOFs were immersed in phosphate buffered saline (PBS), framework degradation led to decarbonylation. The amounts of CO released indicated that only 9–17% of CUS in the parent MOFs were occupied by CO, which may have been due to the access of CO molecules to internal CUS being kinetically hindered by the relatively narrow micropores of MIL-88B. Other research in this area involved the noncovalent encapsulation of manganese, rhenium, and molybdenum tricarbonyl complexes in mesoporous materials, layered materials, and MOFs.^20−25^ Mixed results were obtained with respect to the capacity of the carriers to retain the CORMs and/or iCORMs while incubated in biological buffer solutions. For example, an Al-doped mesoporous silica (Al-MCM-41) retained ca. 86% of the cationic complex [Mn(1,4,7-triazacyclononane)(CO)_3_]^+^ after 72 h incubation in PBS,^21^ while alkanesulfonate-functionalized MCM-41 retained only 14% of the same complex after 1 h.^22^ Covalent anchoring of CORMs onto porous materials could be one approach to preventing metal leaching but has not yet met with success. For example, the Zr^IV^ MOF UiO-67-bpydc containing 5,5′-dicarboxylate-2,2′-bipyridine (bpydc) organic linkers was treated with [MnBr(CO)_5_] to obtain photoactive [MnBr(bpydc)(CO)_3_] species on the MOF walls.^26^ Immersion of the material in PBS led to framework decomposition and almost 50% Mn leaching.

Considering the strategies pursued so far to develop inorganic or metal–organic CORMAs, the ideal material might be one in which the CO-releasing fragments form an integral part of the scaffold, i.e., already come “built-in” in the as-prepared material. The benefits of such a CO-delivery system could include a high CO payload, monometallic nature, and good resistance to metal leaching. For these reasons, we became interested in a recent report that described the direct reaction of Group 6 metal hexacarbonyls with pyrazine (pyz) to give crystalline pyz-pillared MOFs with the formula M(CO)_3_(pyz)_3/2_·*n*pyz.^27^ With M = Mo, a triclinic phase (*n* = 0.5) along with a small amount of a cubic phase (*n* = 0) were obtained. These materials hold potential as CORMAs due to the presence of pyz fragments as core components in many approved and investigational drugs,^28^ the favorable safety profile of pyz and derivatives as food additives,^29^ the essential role played by molybdenum as a cofactor for several enzymes in human metabolism,^30,31^ the low toxicity of molybdenum metabolites,^32−34^ and the proven efficacy of molecular tricarbonyl complexes as CORMs.^33,34^ In the present work, we set out to optimize individual syntheses of the two crystalline *fac*-Mo(CO)_3_(pyz)_3/2_ phases. Once this was achieved, we were able to evaluate the CO-release behavior of the MOFs, showing that they behave as CORMAs. These findings were supplemented by studies to compare the stabilities of the materials toward air and aqueous media.

## 2 Experimental Section

### 2.1 Materials

The following chemicals, reagents, and solvents were obtained from commercial sources and used as received: (for synthesis) molybdenum hexacarbonyl (Fluka, Buchs, Switzerland), pyrazine (>99%, Sigma-Aldrich, Merck Life Science S.L.U., Portugal), toluene (99.9%, Sigma-Aldrich), acetonitrile (99.9%, Riedel-de Haën, Seelze, Germany); (for the myoglobin assay) Mb from equine skeletal muscle (95–100%, lyophilized powder), sodium dithionite, and HEPES buffer (99.5%) were all obtained from Sigma-Aldrich. The gases used in this work were carbon monoxide (≥ 99.0% purity, Sigma-Aldrich) and nitrogen (Alphagaz Nitrogen type 1, 99.999% (H_2_O < 3 ppm, O_2_ < 2 ppm), AirLiquide, Algés, Portugal).

### 2.2 MOF Syntheses

#### 2.2.1 *fac*-Mo(CO)_3_(pyz)_3/2_·1/2 pyz (**Mo-hex**)

The procedure described by Voigt et al. was followed, using the same reagent quantities and ampoule volume (20 mL).^27^ A scaled-up synthesis was subsequently carried out using twice the reagent quantities and an ampoule volume of 40 mL. In the small-scale synthesis, Mo(CO)_6_ (0.08 g, 0.30 mmol) and pyz (52 equiv) were added to the glass ampoule, which was evacuated for 2 min to remove all oxygen, and then immediately flame sealed. The ampoule was placed in an oven, heated to 150 °C at a ramp rate of 0.5 °C min^–1^ and left at that temperature for 40 h. Afterward, the ampoule was cooled to room temperature over 16–24 h, and the black shiny crystalline product was transferred to a Schlenk tube and washed with acetonitrile (3 × 15 mL) to remove excess pyz and residual Mo(CO)_6_. Finally, the solid was vacuum-dried at room temperature for 2 h, obtaining the material **Mo-hex** (0.06 g, 59% based on Mo). The same relative yield was obtained with the large-scale synthesis (0.12 g, 59% based on Mo). Anal. calcd for C_9_H_6_MoN_3_O_3_.C_2_H_2_N (340.15): C, 38.84; H, 2.37; N, 16.47%. Found for small-scale: C, 38.94; H, 2.44; N, 16.70%. Found for large-scale: C, 39.11; H, 2.47; N, 16.67%. TGA under air showed a residual mass of 42.5% at 400 °C (calcd for MoO_3_: 42.3%).

#### 2.2.2 *fac*-Mo(CO)_3_(pyz)_3/2_ (**Mo-cub**)

An open reflux method using toluene as solvent yielded the pure cubic phase formulated as *fac*-Mo(CO)_3_(pyz)_3/2_ (**Mo-cub**). The reaction time (1–3 h) and amount of pyz (6–52 equiv) were varied to optimize product crystallinity and yield (Figure S1 in the Supporting Information). In the optimized small-scale synthesis, a mixture of Mo(CO)_6_ (0.16 g, 0.60 mmol) and pyz (10 equiv) was evacuated for 10 min in a Schlenk tube, and then toluene (10 mL) was added and the mixture was refluxed under a nitrogen atmosphere for 3 h. Afterward, the resultant dark precipitate was isolated by centrifugation, washed with acetonitrile (3 × 20 mL), and vacuum-dried at room temperature for 2 h, obtaining the material **Mo-cub** (0.13 g, 72% based on Mo). A good yield of **Mo-cub** (0.41 g, 76%) was also obtained in a scaled-up synthesis performed using a 3-fold increase in the amounts of solvent and reagents. Anal. calcd for C_9_H_6_MoN_3_O_3_ (300.10): C, 36.02; H, 2.01; N, 14.00%. Found for small-scale: C, 35.82; H, 2.23; N, 13.89%. Found for large-scale: C, 36.26; H, 2.09; N, 14.23%. TGA under air showed a residual mass of 49.6% at 400 °C (calcd for MoO_3_: 48.0%).

### 2.3 Characterization Methods

Powder X-ray diffraction (PXRD) patterns were collected on a Malvern Panalytical (Malvern, UK) Empyrean diffractometer (Cu-Kα X-radiation, λ = 1.54060 Å) in a Bragg–Brentano para-focusing optics configuration (45 kV, 40 mA) at ambient temperature, using a spinning flat plate sample holder. Samples were step-scanned in the range from 5 to 70° (2θ) with steps of 0.026°. A PIXEL linear detector with an active area of 1.7462° was used with a counting time of 99 s per step. Variable temperature PXRD was carried out using a Malvern Panalytical X’Pert PRO3 HTK 16 N high-temperature chamber containing a Pt heating filament and a Pt–Pt/Rh (10%) thermocouple. The powdered sample was deposited on a Pt sheet, which was then placed over the heating element. A heating rate of 5 °C min^–1^ was used. At a given temperature, samples were step-scanned in 0.021° 2θ steps with a counting time of 100 s per step. Scanning electron microscopy (SEM) images were obtained on a Hitachi SU-70 SEM microscope (Hitachi High-Tech Europe GmbH, Krefeld, Germany) equipped with a Bruker Quantax 400 detector (Bruker, Billerica, MA, USA) operating at 20 kV. Elemental analysis for C, H, and N was performed using a Truspec 630-200-200 instrument (Leco, Saint Joseph, MI, USA). Thermogravimetric analysis (TGA) was performed under air using a Hitachi STA 300 system with a heating rate of 5 °C min^–1^.

Attenuated total reflectance (ATR) FT-IR spectra were measured on a Bruker Tensor 27 spectrometer equipped with a Specac Golden Gate Mk II ATR accessory (Specac, Orpington, UK) having a diamond top plate and KRS-5 focusing lenses (resolution 4 cm^–1^, 256 scans). FT-Raman spectra were recorded on a Bruker MultiRAM spectrometer equipped with a Nd:YAG laser with an excitation wavelength of 1064 nm. Solid-state ^13^C{^1^H} cross-polarization (CP) magic-angle spinning (MAS) NMR spectra were recorded using a Bruker Avance 400 spectrometer with an ultrashielded static magnetic field of 125.76 MHz. The spectra were recorded with 3.2 μs pulses, 3.5 ms contact time, spinning rate of 12 kHz, and 5 s recycle delays. Solution ^13^C{^1^H} NMR spectra were acquired at 75.47 MHz with a Bruker Avance 300 spectrometer. The solution UV–vis spectra were collected using a GBC 918 spectrophotometer (GBC Scientific Equipment, Australia).

### 2.4 CO-Release Assays

CO release from the MOFs **Mo-cub** and **Mo-hex** was quantified by the myoglobin (Mb) assay,^35^ which uses absorption spectroscopy to follow the conversion of deoxy-Mb to carbonmonoxy-myoglobin (MbCO). The formation of MbCO is evidenced by spectral changes in the Q-band region (540–580 nm) and/or by the shift of the Soret band from 433 to 423 nm. Interference from free pyrazine in solution (which could conceivably arise from leaching of the noncoordinated pyz molecules in **Mo-hex**) may be discounted on the basis that native ferrous Mb does not bind pyridine.^36^

Stock solutions/suspensions of Mb (100 μM), sodium dithionite (40 mg/mL), and **Mo-cub** or **Mo-hex** (0.60–0.68 mg/mL, 2 μmol_Mo_/L) were prepared in N_2_-degassed 10 mM HEPES (pH 7.4). Under a nitrogen atmosphere, the stock solutions were added to a sealed semimicro (3500 μL) quartz cuvette in the following order: 2385 μL of 10 mM HEPES, 300 μL of Mb (100 μM), 300 μL of sodium dithionite (40 mg/mL), and 15 μL of MOF suspension (2 mM). The sample was incubated in the dark at 37 °C with magnetic stirring at 400 rpm. The assay was conducted over 24 h, interrupting the incubation in intervals of 60 min for the first 6 h to collect an absorption spectrum of the sample between 400 and 700 nm, with a scan speed of 200 nm/min and a slit width of 2 nm. At the end of the final incubation period, the sample was converted to 100% MbCO by bubbling CO gas through the liquid phase for 5 min, and an absorption spectrum was recorded. The actual concentration of Mb in the cell ([Mb] = 6.6–8.1 μM) was then determined by using an extinction coefficient of 207 mM^–1^ cm^–1^ (MbCO) at 423 nm.^37^ All the assays were carried out in triplicate, and the spectroscopic data were treated in the standard way by applying a correction at the 510 nm isosbestic point.^38^

Mb assays were subsequently performed under photoexcitation using either a 15 W Velleman lamp (ref LAE1F8F) with λ = 365 nm (24 h assay, 37 °C) or a Fluorolog-3 Horiba Scientific (Horiba Scientific, Kyoto, Japan) spectrofluorometer (Model FL3-2 T) equipped with a 450 W xenon lamp and capable of double excitation (365 nm and 506 nm, 22 °C, 3 h). The UV lamp and Fluorolog Model powers were measured with a Coherent FieldMaxII-TOP power sensor (Coherent, Barcelona, Spain), revealing irradiance values of 6.5 and 610 mW cm^–2^, respectively.

### 2.5 Stability Studies

To verify the stability of the materials in solution, 20 mg of **Mo-cub** or **Mo-hex** was added to N_2_-degassed 10 mM HEPES (20 mL, pH 7.4). The suspensions were magnetically stirred at 37 °C for 24 h to mimic the simulated physiological conditions of the Mb assay. After this incubation period, the solids (denoted **Mo-cubL** and **Mo-hexL**) were separated from the mother liquors, vacuum-dried, and characterized by PXRD and ATR FT-IR spectroscopy.

## 3 Results and Discussion

### 3.1 Synthesis and Characterization of *fac*-Mo(CO)_3_(pyz)_3/2_ MOFs

**Figure 1:**
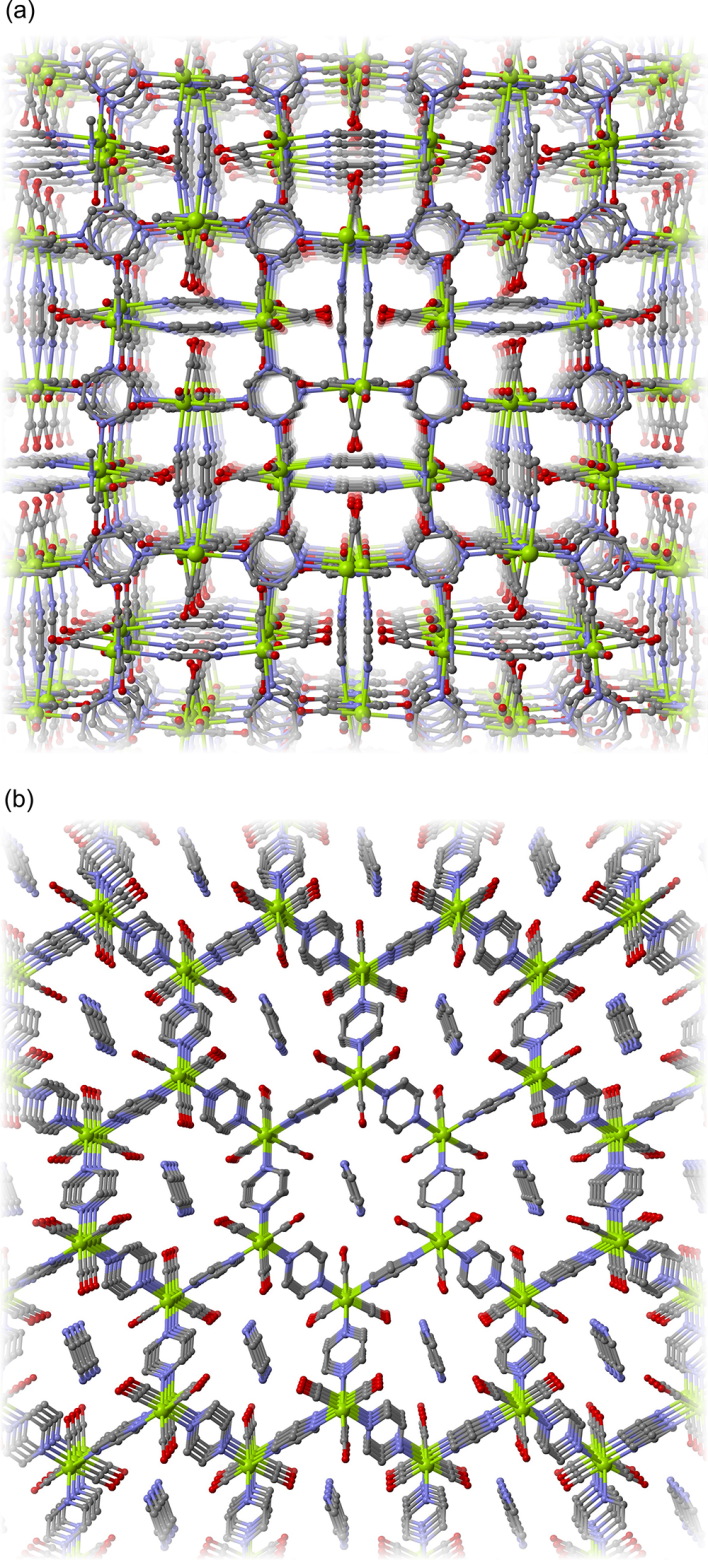
Views of the crystal structures of (a) **Mo-cub** and (b) **Mo-hex**. Hydrogen atoms have been omitted for clarity. Mo, green; carbon, gray; nitrogen, blue; oxygen, red.

Reaction of pyz with Mo(CO)_6_ in sealed ampoules at 150 °C gave dark crystalline products with PXRD patterns that matched that reported by Voigt et al. (Figure S2 in the Supporting Information).^27^ Structure elucidation by single-crystal X-ray diffraction identified the major crystalline phase of this solvent-free reaction as the MOF with the formula *fac*-Mo(CO)_3_(pyz)_3/2_·1/2 pyz (**Mo-hex**).^27^ In the structure of **Mo-hex**, which crystallizes in the triclinic space group *P*1̅, the stacking of *fac*-Mo(CO)_3_(pyz)_3/2_ coordination layers along the *a*-axis generates narrow hexagonal pore channels that house noncoordinated pyz molecules (Figure 1b). The PXRD pattern of the bulk crystalline product obtained in the solvent-free reaction contains some reflections (identified with asterisks in Figure S2) that are due to a minor secondary crystalline phase. Although Voigt et al. did not report a method to prepare this second phase in a pure form, the mechanical separation of single crystals allowed the compound to be identified by XRD as a cubic phase with the formula *fac*-Mo(CO)_3_(pyz)_3/2_ (**Mo-cub**). **Mo-cub** possesses a dense framework made up of two interpenetrating coordination networks with *fac*-Mo(CO)_3_(pyz)_3/2_ building units (Figure 1a). We found that crystalline **Mo-cub** could be obtained in a pure form by a solvothermal method in which Mo(CO)_6_ and an excess of pyz are reacted in refluxing toluene. The PXRD pattern for the solid product obtained by this method matches very well with the simulated pattern calculated from the crystal structure (Figure S1 in the Supporting Information).^27^ The reflux method is fast (3 h), reproducible and scalable, requires a relatively small 10-fold excess of pyz, and gives good yields of **Mo-cub** (72–76%). In contrast, the ampoule method requires a longer reaction time (40 h) and a 50-fold excess of pyz, and the MOF yield in our experiments was consistently around 59%. These results suggest that the synthesis kinetics was enhanced by the reflux method, which is at least partly due to the fluid dynamics favoring mass transfer.

**Figure 2:**
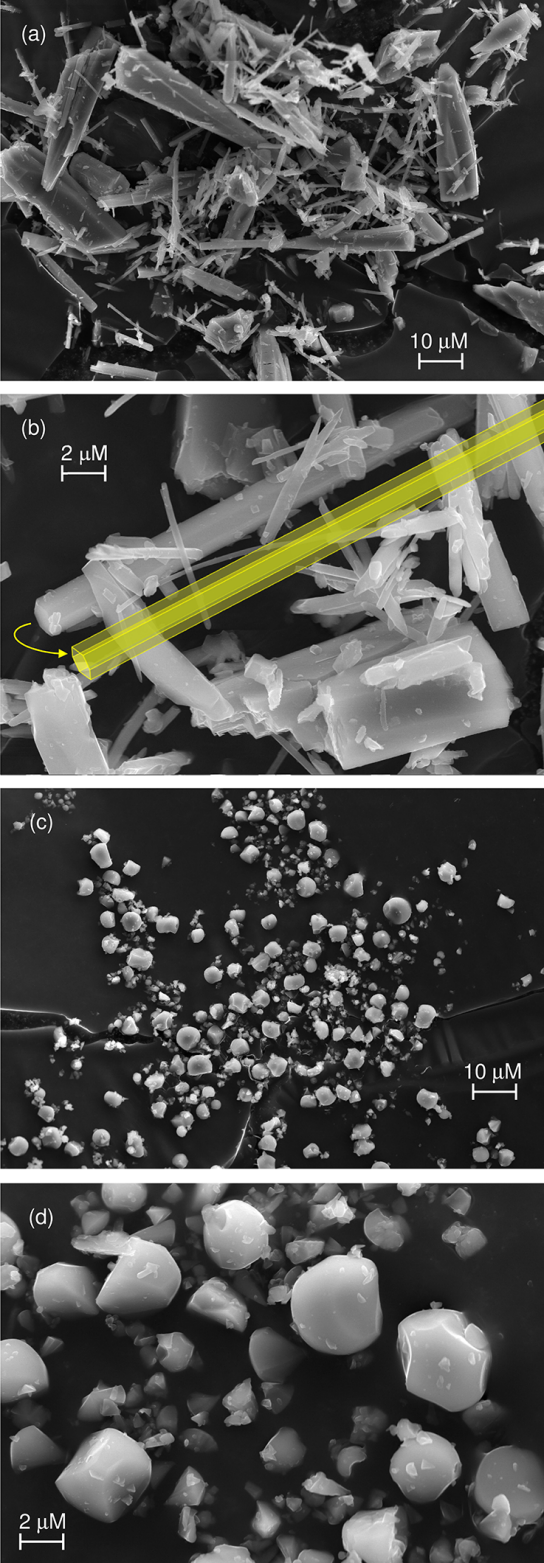
Representative SEM images at different magnifications for (a, b) **Mo-hex** and (c, d) **Mo-cub**.

Crystal morphologies were assessed by SEM (Figure 2). **Mo-hex** shows an irregular rod-like or needle-like morphology with particle sizes up to 30 μm, with some showing a clearly defined hexagonal rod-like character (highlighted in Figure 2b). The particles of **Mo-cub** generally present an irregular and pseudo-spherical aspect with sizes up to 5 μm, with a small number of particles showing a more defined, albeit fragmented, shape resembling a truncated octahedron.

**Figure 3:**
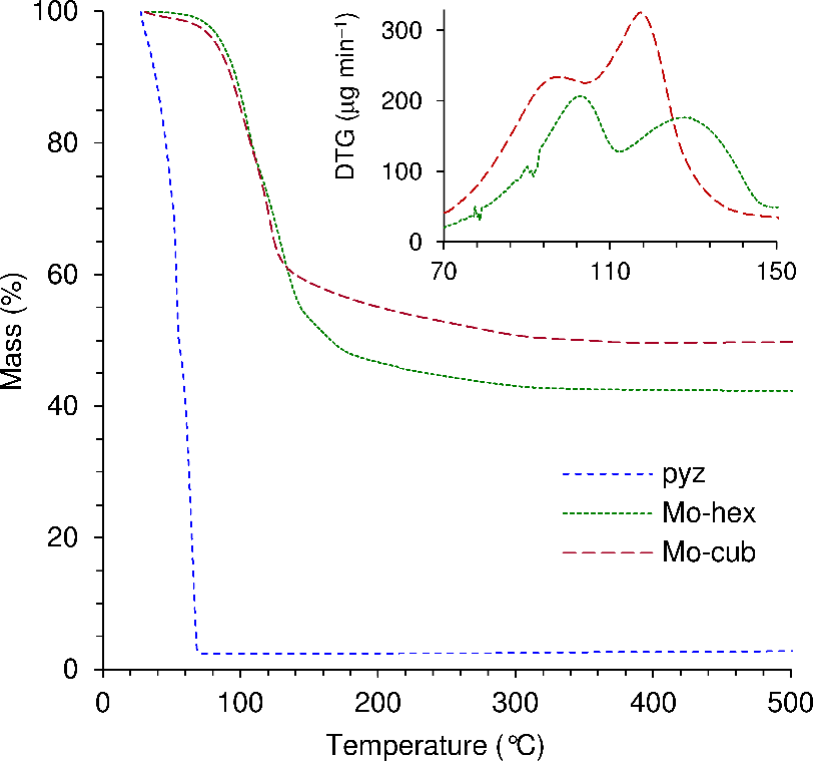
TGA curves (under air) of pyz, **Mo-hex**, and **Mo-cub**. The inset shows the DTG profile for **Mo-hex** and **Mo-cub** between 70 and 150 °C.

**Figure 4:**
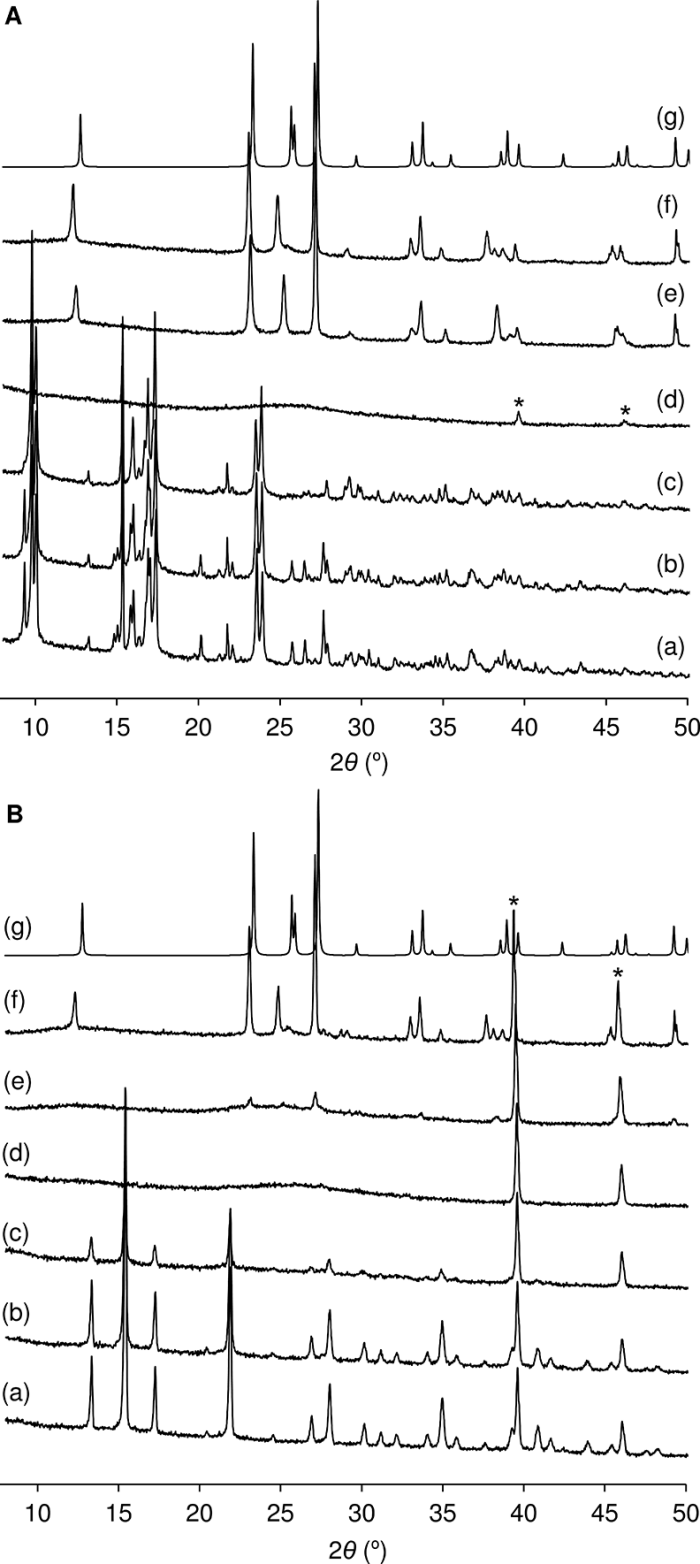
Variable temperature PXRD study of (**A**) **Mo-hex** and (**B**) **Mo-cub**: (a) 25, (b) 40, (c) 65, (d) 150, (e) 320, and (f) 600 °C. Pattern (g) is a simulated pattern for α-MoO_3_ calculated using the program Mercury.^39^ The asterisks mark reflections due to the platinum sample holder.

The thermal stability of the materials was evaluated by TGA (Figure 3) and variable-temperature PXRD (VT-PXRD) (Figure 4). For comparison, the TGA curve of neat pyz is shown. Pyz readily sublimes at room temperature and atmospheric pressure, as shown by the abrupt and complete mass loss between 25 and 70 °C (differential thermogravimetric maximum (DTG_max_) at 66 °C). Although **Mo-hex** contains free pyz molecules in the hexagonal pore channels, the TGA curve of the MOF under air does not show an early (<70 °C) weight loss step corresponding to the removal of these weakly bound moieties. Instead, **Mo-hex** starts to decompose above 70 °C, showing two main overlapping weight loss steps with DTG_max_ values of 103 and 128 °C. The first of these is probably due to partial decarbonylation combined with removal of the free pyz molecules, while the second step is attributed to decomposition of the remaining pyz molecules in the framework. **Mo-cub** displays a similar behavior with DTG_max_ values of 97 and 118 °C. The weight of the residue at 400 °C for both MOFs is consistent with α-MoO_3_ being the final product. The observed (calculated) values are 42.5 (42.3%) and 49.6% (48.0%) for **Mo-hex** and **Mo-cub**, respectively.

In line with the TGA profiles, the in situ VT-PXRD study showed that the reflections characteristic of the triclinic phase (Figure 4A) and cubic phase (Figure 4B) are present up to about 65 °C. Heating to 150 °C results in loss of crystallinity to give an amorphous phase with only one very broad peak between 20 and 30° 2θ. In each case, at 320 °C, new reflections appear that match those present in the simulated PXRD pattern for orthorhombic α-MoO_3_ (Figure 4g). The molybdenum oxide phase persists up to 600 °C. The in situ VT-PXRD study did not show interconversion between the triclinic and cubic phases, nor the appearance of new phases other than α-MoO_3_.

**Figure 5:**
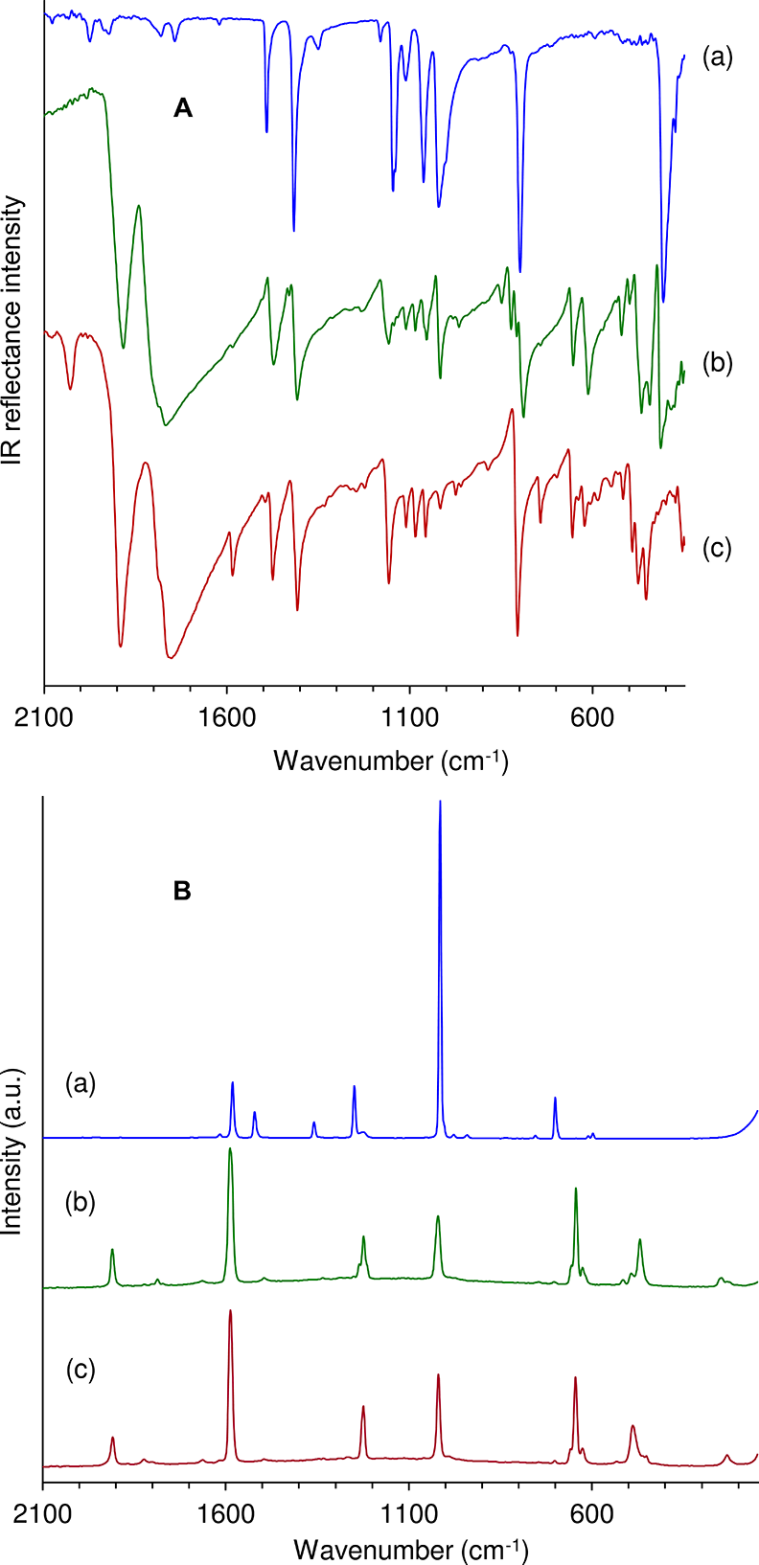
ATR FT-IR (**A**) and FT-Raman (**B**) spectra of (a) pyz, (b) **Mo-hex**, and (c) **Mo-cub**.

Figure 5 shows the ATR FT-IR and FT-Raman spectra of **Mo-hex** and **Mo-cub** in the spectral ranges 350–2100 and 180–2100 cm^–1^, respectively. For comparison, the spectra of pyz are shown. Both MOFs display two strong ν(CO) IR bands near 1890 and 1760 cm^–1^ consistent with the A_1_ and E modes expected for a pseudo *C*_3v_ local symmetry and hence a facial arrangement of the carbonyl groups.^40−42^ The lower-energy band is broadened and split, with a shoulder around 1785 cm^–1^, which may stem from solid-state effects.^41,42^ The A_1_ mode gives rise to a sharp band around 1910 cm^–1^ in the Raman spectra. The IR spectra for **Mo-cub** reproducibly display an additional weak band at 2029 cm^–1^. This band, which lies within the carbonyl stretching region but is unexpected for *fac*-Mo(CO)_3_L_3_ coordination structures, has not yet been satisfactorily assigned.

For **Mo-hex** and **Mo-cub**, the vibrational bands observed in the 180–1600 cm^–1^ region are due to internal pyz vibrations, metal–carbon stretching [ν(MC)], metal–carbon–oxygen bending [δ(MCO)], and metal–ligand [ν(MN)] modes of vibration. The vibrational spectrum of pyz has been the subject of several studies.^43−46^ The pyz absorptions in the range 350–1600 cm^–1^ arise from ring stretching, CH/ring in-plane and out-of-plane bending modes. Coordination of pyz to metal centers affects these bands to varying degrees. Of particular use for diagnostic purposes is the shift of the IR-active out-of-plane ring bending mode near 410 cm^–1^ for free pyz to higher frequencies: to about 450 cm^–1^ for monodentate (terminal) coordination and to 470 cm^–1^ or higher for bidentate bridging coordination.^47,48^ For the MOFs **Mo-hex** and **Mo-cub**, the assignment of bands in the range 400–500 cm^–1^ to pyz is difficult because ν(MC) modes for Group 6 metal carbonyls occur within the same frequency range.^40,49−52^ An added complication is the presence of δ(MCO) modes in the low-frequency region, although these modes usually occur at higher frequencies (500–700 cm^–1^) than ν(MC) modes.^50−52^ The 400–700 cm^–1^ region of the ATR FT-IR spectrum of **Mo-hex** contains at least seven bands with medium to strong intensity. The band at 415 cm^–1^ is assigned to uncoordinated pyz molecules located in the hexagonal channels. One weak (499 cm^–1^) and two medium intensity (444 and 466 cm^–1^) bands are observed in the 420–500 cm^–1^ region. Realistically, it is not possible to distinguish between ν(MC) and pyz-centered assignments for these bands. Assuming that the lower frequency band is not due to monodentate (terminal) pyz molecules, the most likely assignment is ν(MoC), although an assignment to bidentate pyz molecules cannot be completely excluded since bands at this frequency and assigned to pyz have been observed, for example, for copper(I) polymer frameworks possessing symmetrically bridging coordinated pyz.^53,54^ The 420–500 cm^–1^ region of the ATR FT-IR spectrum of **Mo-cub** is like that for **Mo-hex** in that three bands are observed at 454, 476, and 492 cm^–1^, which are tentatively assigned to ν(MoC), pyz ring deformation, and δ(MCO). No band occurs in the 400–420 cm^–1^ region, confirming the absence of uncoordinated pyz molecules. On the other hand, some features of the spectrum of **Mo-cub** indicate the presence of monodentate (terminal) pyz moieties. A medium intensity band at 1585 cm^–1^ is assigned as a centrosymmetric ring stretching mode.^43−46,55^ This mode is Raman active for terminal monodentate, bridging bidentate, and free ligand (a medium intensity band is found at 1582 cm^–1^ in the Raman spectrum of free pyz, and both MOFs display a very strong band at 1588 cm^–1^) but only IR active for the terminal coordination mode.^55^ Hence, the occurrence of this band with medium intensity in the IR spectrum of **Mo-cub** suggests the presence of a substantial number of monodentate pyz ligands, possibly concentrated at the exterior surface of the MOF crystals.^55^**Mo-hex** evidently contains a much lower proportion of these groups since only a very weak band is observed at the same frequency. Concordantly, a band at 743 cm^–1^ appears with weak intensity for **Mo-hex** and medium intensity for **Mo-cub**. As noted by Haynes et al.,^47^ the presence of an IR band at approximately 750 cm^–1^ is another criterion for the existence of unidentate pyz groups in metal coordination compounds. This band, which has been assigned to an out-of-plane ring bending vibration,^44^ appears with weak intensity at 746 cm^–1^ in the Raman spectra of the two MOFs.

The Raman spectra of **Mo-hex** and **Mo-cub** are very similar in the range 600–2100 cm^–1^. Other than the two bands for ν(CO) and ν(ring) mentioned above, the main bands in this region are δ(MCO) at 645 cm^–1^, ν(ring) at 1020 cm^–1^, and δ(CH) at 1224 cm^–1^. In the low frequency region, a medium intensity band at 469 cm^–1^ for **Mo-hex** and 488 cm^–1^ for **Mo-cub** is assigned to ν(MoC), and a weaker band at 246 cm^–1^ for **Mo-hex** and 231 cm^–1^ for **Mo-cub** is assigned to ν(MoN).^52^

**Figure 6:**
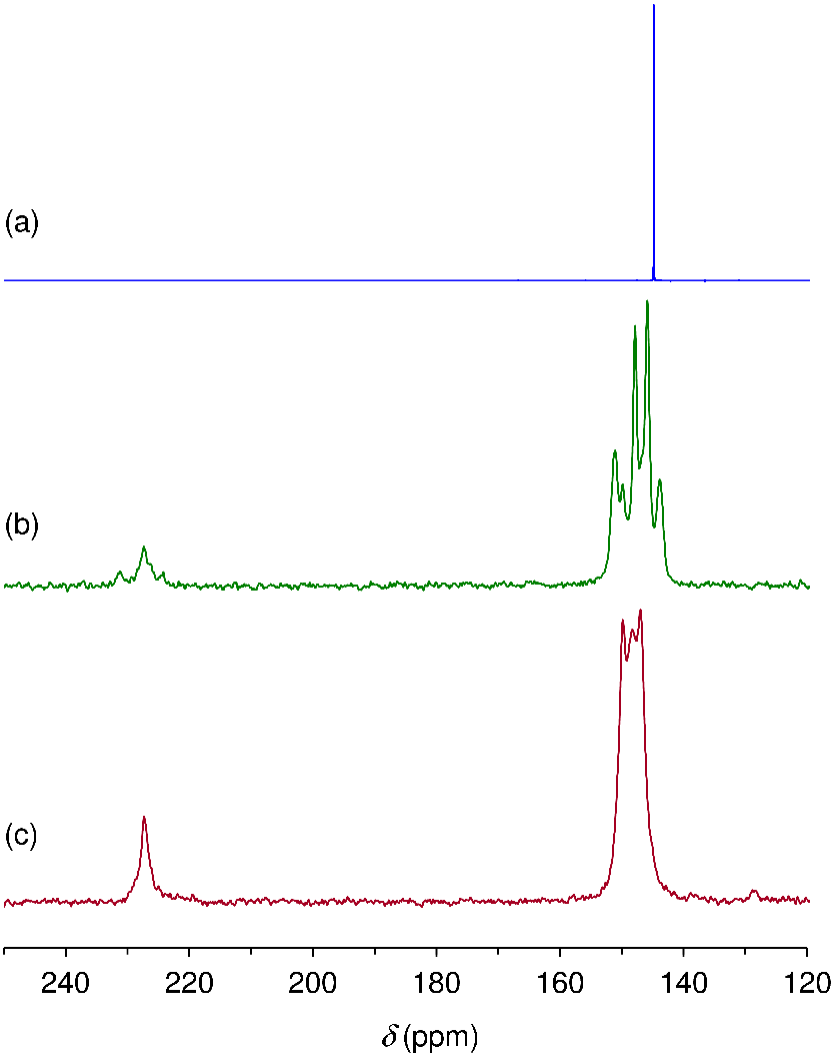
^13^C{^1^H} CP MAS NMR spectra of (b) **Mo-hex** and (c) **Mo-cub** compared with (a) the solution ^13^C{^1^H} NMR spectrum of pyrazine (CDCl_3_).

The ^13^C{^1^H} CP MAS NMR spectra of **Mo-hex** and **Mo-cub** are shown in Figure 6. **Mo-cub** displays a single relatively sharp resonance for the carbonyl groups at δ = 227 ppm, which agrees with the crystallographic equivalence of the three Mo-coordinated CO groups in the cubic phase.^27^ On the other hand, while only two resonances are expected for the pyz carbon atoms (since the three Mo-coordinated pyz groups are also crystallographically equivalent, with each ring providing two pairs of crystallographically unique but chemically equivalent carbons), three resolved lines are observed at δ = 147.0, 148.3, and 149.8 ppm. This may be due to solid-state effects and/or (as discussed above) the presence of a significant proportion of unidentate (terminal) pyz groups in addition to the symmetrically bridging ligands. The ^13^C{^1^H} CP MAS NMR spectrum of **Mo-hex** is more complex, showing at least six distinct pyz resonances in the range of 144–151 ppm and three carbonyl resonances between 224 and 231 ppm. This is in accordance with the crystal structure, which shows eight crystallographically distinct pyz carbons (two from the free pyz molecule and two from each of the three Mo-coordinated pyz ligands) and three inequivalent carbonyl groups.

The stability of the MOFs toward air was evaluated by recording PXRD patterns and ATR FT-IR spectra as a function of exposure time (Figure S3 in the Supporting Information). PXRD showed retention of the crystalline **Mo-hex** phase after 45 days of exposure to air inside a closed Eppendorf vial. Slight alterations were observed in the IR spectra, specifically regarding the appearance of broad bands at approximately 730 and 964 cm^–1^. In contrast to **Mo-hex**, **Mo-cub** showed significant loss of crystallinity after two weeks of exposure, accompanied by the formation of an amorphous phase (very broad reflection centered at approximately 7.0° 2θ). The ATR FT-IR spectrum of **Mo-cub** showed alterations after 2 weeks, including the appearance of a broad band at 958 cm^–1^, similar to that seen for **Mo-hex**, and the disappearance of the band at 2029 cm^–1^. On the other hand, even after 30 days of exposure to air, the IR spectrum of **Mo-cub** still exhibited very strong ν(CO) bands at 1765 and 1895 cm^–1^, similar to those displayed by the pristine as-prepared sample. The new band around 960 cm^–1^ for the two MOFs exposed to air may arise from a ν(Mo=O) vibration and therefore could be a marker for the formation of oxomolybdenum species. Both **Mo-hex** and **Mo-cub** degrade much faster when left in open air. After 10 days, **Mo-cub** underwent almost complete decarbonylation (shown by the loss of the ν(CO) bands) and transformed to an amorphous phase, which exhibited three main broad absorption bands in the IR spectrum at 710, 960, and 1614 cm^–1^ (Figure S3D). These three bands also appeared for **Mo-hex** after 10 days of exposure (Figure S3C in the Supporting Information). However, rather than undergoing complete decarbonylation to give an amorphous phase, ν(CO) bands at 1770 and 1884 cm^–1^ were retained, albeit with diminished intensity, and the PXRD pattern displayed sharp reflections, although differences were observed vs the pattern for as-prepared **Mo-hex**, mainly concerning the loss of some reflections or changes in relative intensities.

### 3.2 CO-Release from *fac*-Mo(CO)_3_(pyz)_3/2_ MOFs

**Figure 7:**
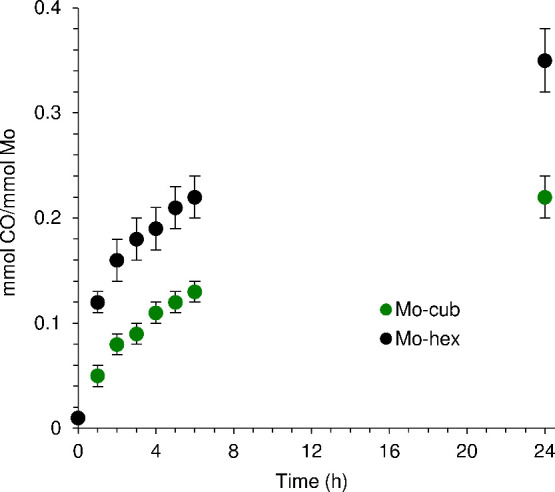
Time courses of CO release (measured by the Mb assay) from **Mo-cub** and **Mo-hex** in the dark when suspended in 10 mM HEPES at 37 °C.

CO-release from the materials **Mo-cub** and **Mo-hex** was investigated under simulated biological conditions (10 mM HEPES buffer, pH 7.4, 37 °C), first in the dark, using the deoxy-myoglobin (deoxy-Mb)/carbonmonoxy-myoglobin (MbCO) UV–vis assay. A degassed HEPES solution of Mb was freshly reduced with excess sodium dithionite under a nitrogen atmosphere, and then the MOF was added. Spectral changes in the Soret band region, namely, the loss of the band due to deoxy-Mb (λ_max_ at 433 nm) and the appearance of the band due to MbCO (λ_max_ at 423 nm), indicated the loss of CO from the MOFs. The decarbonylation reached 0.22 mmol CO per mmol of molybdenum (0.64 mmol CO per gram of material) for **Mo-cub** and 0.35 mmol CO per mmol of molybdenum (1.02 mmol CO per gram of material) for **Mo-hex** after 24 h of incubation (Figure 7). The total amount of CO released did not reach a constant value (i.e., plateau) for either material within 24 h, demonstrating a slow and sustained release profile.

Having determined that **Mo-hex** and **Mo-cub** slowly release relatively small amounts of CO in the dark under the simulated biological conditions of the Mb assay, assays were subsequently performed with irradiation of the samples using a low power UV lamp (λ = 365 nm) to establish whether CO-release from the materials was photoactivatable. Comparison of the Mb assays performed over 24 h at 37 °C in the dark or under irradiation showed no significant differences regarding the CO-release profiles (Figure S4 in the Supporting Information). Additional photochemical studies were conducted at two different wavelengths (365 and 505 nm) using the 450 W xenon lamp of a spectrofluorometer as the excitation source, with a power density of 610 mW cm^–2^ (ca. 100-fold higher than that measured for the low power UV lamp). Mb assays performed for **Mo-cub** at 22 °C for 3 h led to the release of 0.21 mmol CO per mmol of molybdenum with irradiation at 365 nm and 0.22 mmol CO per mmol of molybdenum with irradiation at 505 nm. A control assay performed in the dark under the same conditions (22 °C) resulted in the release of 0.18 mmol CO per mmol of molybdenum. The minor differences observed between the assays carried out in the dark or under low/high power irradiation suggest that the MOFs are photostable and that neither an increase in the incident light intensity nor a change in the wavelength (in the interval studied) induce photodecarbonylation.

To date, most CORMAs have required irradiation with UV or visible light to trigger the release of CO. Spontaneous CO-release from MOF-based CORMAs under physiological conditions (i.e., not requiring a specific trigger such as light) has only been previously reported for CO-loaded iron-carboxylate materials with the MIL-88B framework-type, which released CO upon degradation of the frameworks in aqueous phosphate buffer at pH 7.4 and 37 °C.^18^ This kind of spontaneous CO release, which typically occurs via ligand exchange/substitution or hydrolysis, may be convenient or even essential in situations where the use of internal (e.g., an enzymatic reaction) or external (e.g., photoactivation) stimuli for CO release is not possible. Metzler-Nolte and co-workers reported maximum CO-release amounts of 0.36 and 0.69 mmol CO per gram of CO-loaded MIL-88B-Fe and NH_2_-MIL-88B-Fe, respectively, with half-lives of 38 and 76 min (measured by the Mb assay).^18^ Hence, the amount of CO released from **Mo-cub** under physiological conditions was similar to that found for the NH_2_-MIL-88B-Fe material, while **Mo-hex** released the higher amount of 1.02 mmol CO per gram of material. The half-lives of CO-release from **Mo-hex** (3 h) and **Mo-cub** (4 h) are significantly longer than those found for the iron-based MOFs, indicating lower CO-release rates, which may be beneficial for therapeutic use.

Metzler-Nolte and co-workers determined that the release of CO from the iron-based MOFs was dependent on the degradation of the materials under physiological conditions, which took place over a period of 2–4 h.^18^ To evaluate the stability of the molybdenum tricarbonyl-pyrazine MOFs under the conditions employed for the Mb assay, PXRD patterns and ATR FT-IR spectra were obtained for solids that had been incubated in 10 mM HEPES at 37 °C for 24 h (Figure S6 in the Supporting Information). For **Mo-cub**, practically no change occurred in the IR spectrum after incubation of the solid in HEPES. On the other hand, while PXRD confirmed the continued presence of the cubic phase with only a minor loss of crystallinity (indicated by a slight broadening of the characteristic Bragg reflections), a very broad reflection centered at ca. 8.0° 2θ appeared, indicating the formation of an amorphous phase (cf. the broad reflection observed for solid **Mo-cub** exposed to air). For **Mo-hex**, the treatment in HEPES buffer led to more noticeable changes in the PXRD pattern and IR spectrum, indicating that the triclinic phase is less stable than the cubic phase under these conditions. This may explain why higher amounts of CO were released from **Mo-hex** than from **Mo-cub** under the conditions of the Mb assay. It is notable that the solids recovered after the stability tests in air and in HEPES buffer with **Mo-hex** displayed closely matching PXRD patterns (Figure S5 in the Supporting Information), suggesting that similar structural changes occurred.

## 4 Conclusions

The high CO payload (up to ca. 10 mmol per gram) of the tricarbonyl-pyrazine-molybdenum(0) MOFs **Mo-hex** and **Mo-cub** singles them out as potentially very attractive CORMAs. This view is reinforced by the finding in the present study that the dense cubic phase, Mo(CO)_3_(pyz)_3/2_, can be easily prepared on a large scale by the direct reaction of Mo(CO)_6_ with a moderate excess of pyrazine in refluxing toluene within a few hours. This contrasts with the preparation of the triclinic phase, Mo(CO)_3_(pyz)_3/2_·1/2pyz (**Mo-hex**), which must be performed at 150 °C over a longer period (40 h) in a sealed environment (ampoule), and typically results in variable amounts of **Mo-cub** as a secondary phase. Through the Mb assay, we have confirmed that both MOFs can behave as CORMAs, i.e., transfer CO to heme proteins (heme of Mb), with the trigger being contact of the materials with a physiological buffer. The materials are sufficiently stable under the conditions of the assay to permit a slow release of CO with a half-life between 3 and 4 h. These results are conditioned to some extent by the requirement to perform the Mb assay under an inert atmosphere. Future work will need to employ alternative CO detection methods, such as those based on amperometric CO electrodes, commercial CO detectors, or gas chromatography, that allow the CO-release behavior of the materials to be studied in aerobic aqueous media.

## Supporting Information Available

PXRD patterns of the products obtained from reactions performed to optimize the synthesis of **Mo-cub**, PXRD patterns of **Mo-hex** and **Mo-cub** obtained in the small-scale and large-scale syntheses, PXRD patterns and ATR FT-IR spectra of **Mo-hex** and **Mo-cub** before and after exposure to air or the stability test in HEPES, and time courses of CO release from **Mo-hex** when kept either in the dark or under constant UV irradiation (PDF)
