# Supplementary material for: Tricarbonyl-Pyrazine-Molybdenum(0) Metal–Organic Frameworks for the Storage and Delivery of Biologically Active Carbon Monoxide
Source: ACS Biomater Sci Eng. 2023 Mar 30;9(4):1909–18. doi: 10.1021/acsbiomaterials.3c00140 (PMC10091354; doi:10.1021/acsbiomaterials.3c00140)
Supplement: Supplementary file 1 — ab3c00140_si_001.pdf [file ab3c00140_si_001.pdf]

Supporting Information

Tricarbonyl-Pyrazine-Molybdenum(0) Metal-Organic Frameworks for the Storage and Delivery of Biologically Active Carbon Monoxide

Andreia F. Silva, Isabel B. Calhau, Ana C. Gomes, Anabela A. Valente, Isabel S. Gonçalves,* and Martyn Pillinger*

CICECO - Aveiro Institute of Materials, Department of Chemistry, University of Aveiro, Campus Universitário de Santiago, 3810-193 Aveiro, Portugal

* Correspondence authors.
E-mail: igoncalves@ua.pt (I.S.G), mpillinger@ua.pt (M.P.).

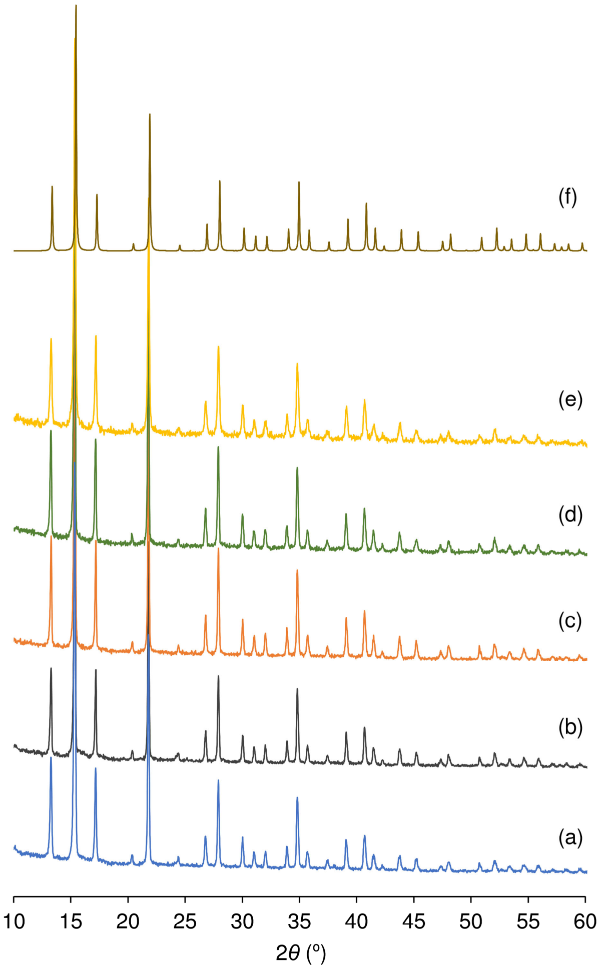

Fig. S1 PXRD patterns of the products obtained from reactions performed to optimize the reaction time and pyz excess for the synthesis of **Mo-cub**: (a) 1 h, 52 eq., (b) 3 h, 52 eq., (c) 3 h, 15 eq., (d) 3 h, 10 eq., and (e) 3 h, 6 eq. Pattern (f) is a simulated pattern calculated using the program Mercury[S1] and the crystal structure data published for **Mo-cub**.[S2]

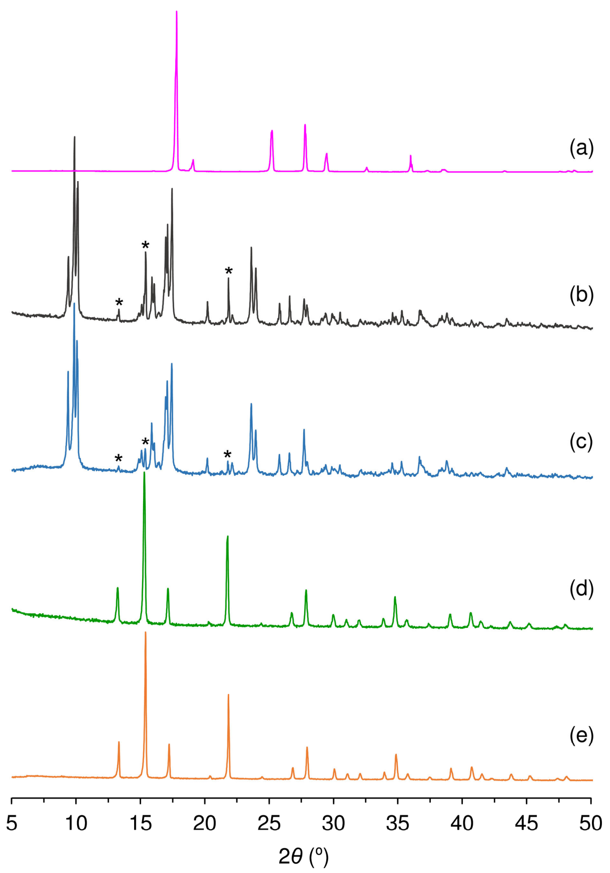

Fig. S2 PXRD patterns of (a) pyrazine, (b) **Mo-hex** (small scale), (c) **Mo-hex** (large scale), (d) **Mo-cub** (small scale), and (e) **Mo-cub** (large scale). Asterisks in patterns (b) and (c) indicate reflections that may be due to **Mo-cub**.

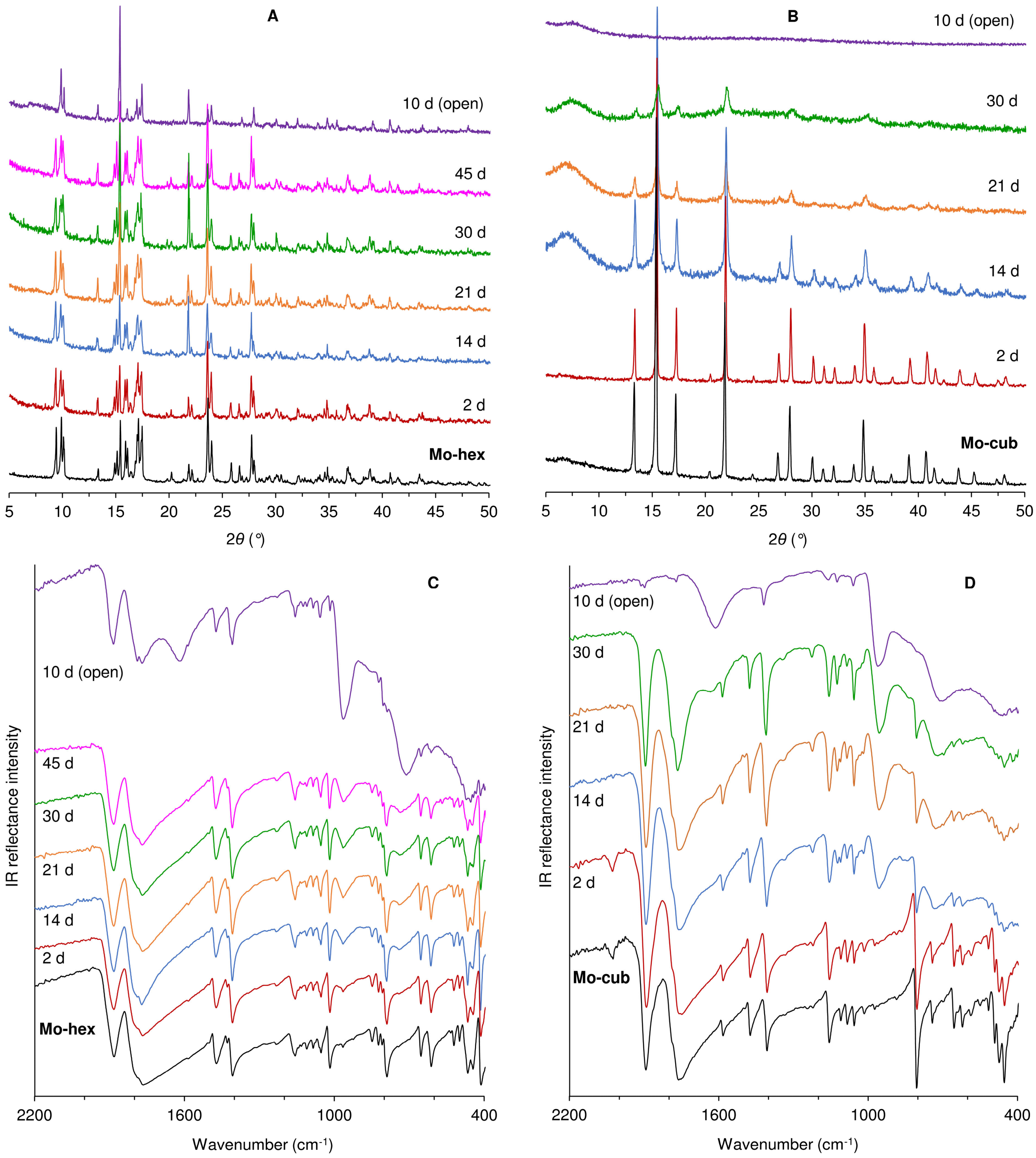

Fig. S3 PXRD patterns (A, B) and ATR FT-IR spectra (C, D) in the range 400-2200 cm^{-1} of **Mo-hex** (A, C) and **Mo-cub** (B, D) before and after 2-45 days of exposure to air in a closed vial at ambient temperature. Data are also shown for 10 days of exposure in open air.

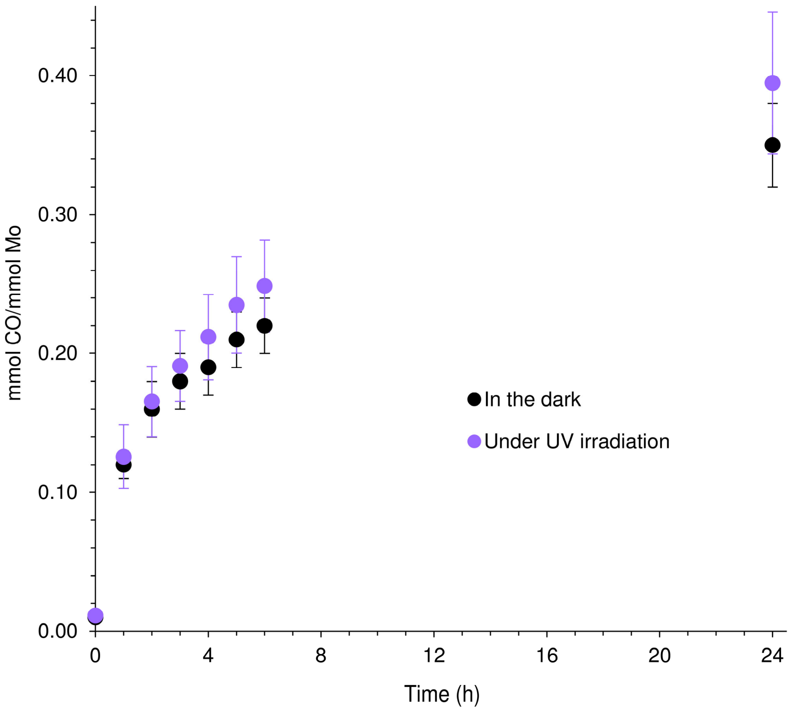

Fig. S4 Time courses of CO release (measured by the Mb assay) from **Mo-hex** when suspended in 10 mM HEPES at 37 °C and kept either in the dark or under constant UV irradiation with a low power UV lamp.

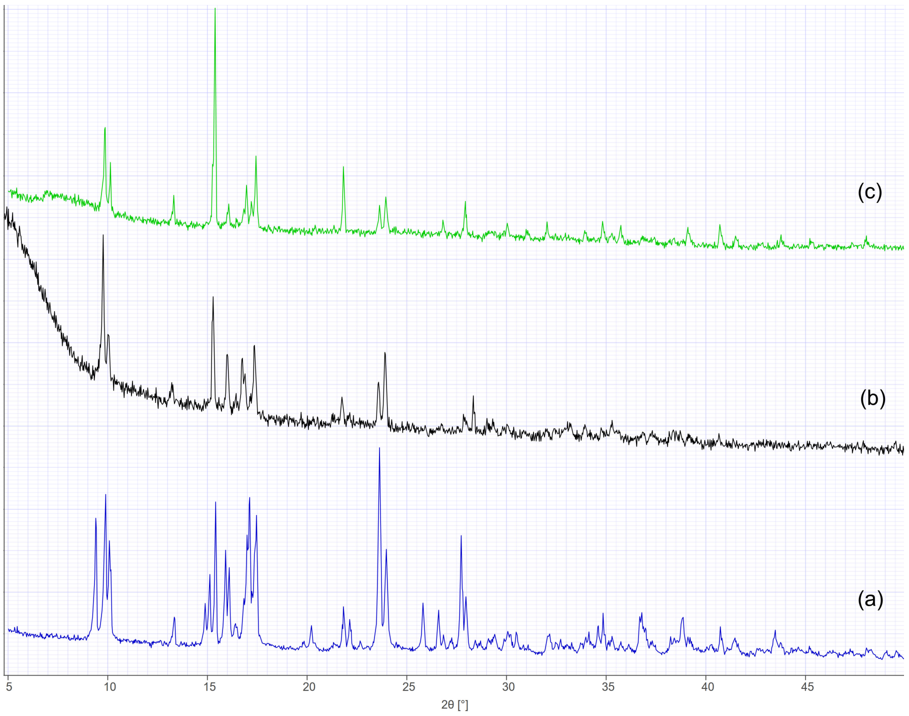

Fig. S5 PXRD patterns of **Mo-hex** before (a) and after (b,c) the stability tests in (b) 10 mM HEPES at 37 °C, and (c) 10 days of exposure of the solid to open air.

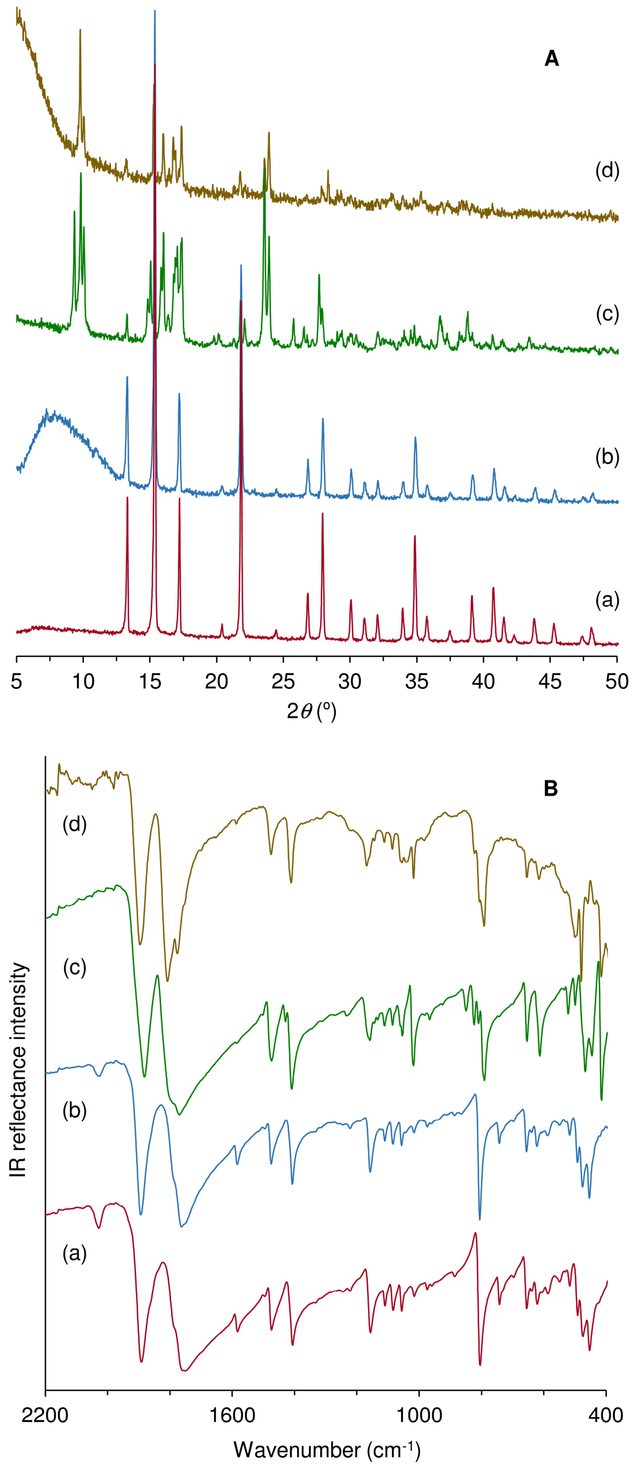

Fig. S6 PXRD patterns (A) and ATR FT-IR spectra (B) in the range 400-2200 cm^{-1} of **Mo-cub** (a,b) and **Mo-hex** (c,d) before (a,c) and after (b,d) the stability test in 10 mM HEPES at 37 °C.

References

S1 C.F. Macrae, I. Sovago, S.J. Cottrell, P.T.A. Galek, P. McCabe, E. Pidcock, M. Platings, G.P. Shields, J.S. Stevens, M. Towler and P.A. Wood, Mercury 4.0: from visualization to analysis, design and prediction, *J. Appl. Crystallogr.*, 2020, **53**, 226-235. https://doi.org/10.1107/S1600576719014092

S2 L. Voigt, R. W. Larsen, M. Kubus and K.S. Pedersen, Zero-valent metals in metal-organic frameworks: *fac*-M(CO)$_3$(pyrazine)$_{3/2}$, *Chem. Commun.*, 2021, **57**, 3861-3864. https://doi.org/10.1039/d1cc00864a
